# Relationship Between Qualitative Differences in Discourse Impairment and Theory of Mind in Patients With Right Hemisphere Damage: A Case Series

**DOI:** 10.7759/cureus.102665

**Published:** 2026-01-30

**Authors:** Kengo Hoyano, Yasutaka Kobayashi

**Affiliations:** 1 Graduate School of Health Sciences, Fukui Health Science University, Fukui, JPN

**Keywords:** case series, discourse impairment, inferential discourse, pragmatic communication, right hemisphere damage, theory of mind

## Abstract

This case series examined qualitative differences in discourse impairment in two elderly right-handed female patients (one in her 60s and another in her 70s) with right hemisphere damage (RHD) and explored the potential involvement of theory of mind (ToM). Patients with RHD often present with discourse-level communication disorders despite relatively preserved basic language abilities. However, the nature of such discourse impairments is not uniform. Discourse performance was assessed using a four-frame cartoon description task, and ToM was evaluated using the Strange Stories test. Case A demonstrated relatively preserved inferential discourse despite making errors in event-related statements, whereas Case B showed prominent impairments in inferential discourse accompanied by marked ToM deficits. These contrasting profiles suggest that discourse impairment following RHD may arise through different underlying mechanisms, with ToM-related processes contributing to inferential discourse disturbances in some individuals.

## Introduction

Right hemisphere damage (RHD) is known to result in communication disorders characterized by reduced discourse coherence, impaired inferential processing, and difficulties in understanding non-literal language, even in the absence of aphasia [1-4]. Such pragmatic impairments can have a substantial impact on everyday communication and social participation [2,3]. Theory of mind (ToM), defined as the ability to infer the mental states of others, plays a crucial role in pragmatic language use [5,6]. Neuroimaging and lesion studies indicate that ToM relies on a distributed network involving the right temporo-parietal junction and medial prefrontal regions [7,8], which may be particularly vulnerable following RHD [9,10]. Previous studies have suggested an association between ToM impairment and pragmatic communication disorders; however, the extent to which ToM contributes to discourse impairment after RHD remains unclear [11-13]. Discourse production requires not only linguistic processing but also the integration of contextual information and inferential reasoning. Importantly, impairments at different processing levels may result in qualitatively distinct discourse profiles [14]. Clarifying such heterogeneity is essential for understanding the mechanisms underlying discourse disorders after RHD. Although discourse impairment following RHD has been widely described, qualitative heterogeneity in discourse profiles and its relationship with ToM remain insufficiently characterized at the case level. Documenting this case series provides clinically grounded evidence that qualitatively distinct discourse impairments may arise from different right-hemispheric mechanisms, underscoring the importance of individualized assessment.

## Case presentation

**Figure 1:**
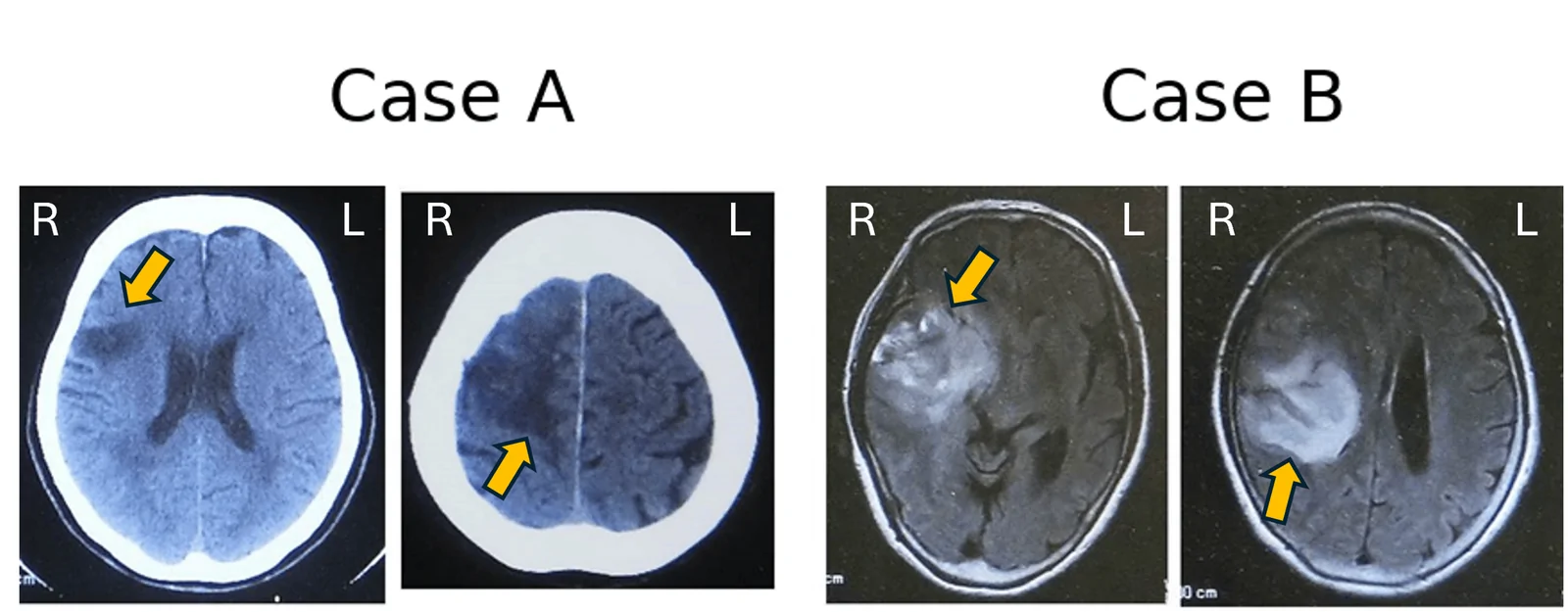
Neuroimaging findings in the two cases Representative axial brain images demonstrating right hemisphere lesions in Case A (computed tomography) and Case B (magnetic resonance imaging).

The two cases were evaluated at a secondary emergency care hospital during the same calendar year following RHD. The cases were identified during routine clinical practice and were not included consecutively. Two right-handed female patients with RHD participated in this study. Case A was a woman in her 60s who experienced a right hemispheric hemorrhagic stroke. Case B was a woman in her 70s who underwent surgical resection of a right hemispheric brain tumor. Neither patient exhibited aphasia, and basic lexical and syntactic abilities were preserved on standard language assessments. Representative neuroimaging findings confirming right hemisphere lesions are shown in Figure 1

**Figure 2:**
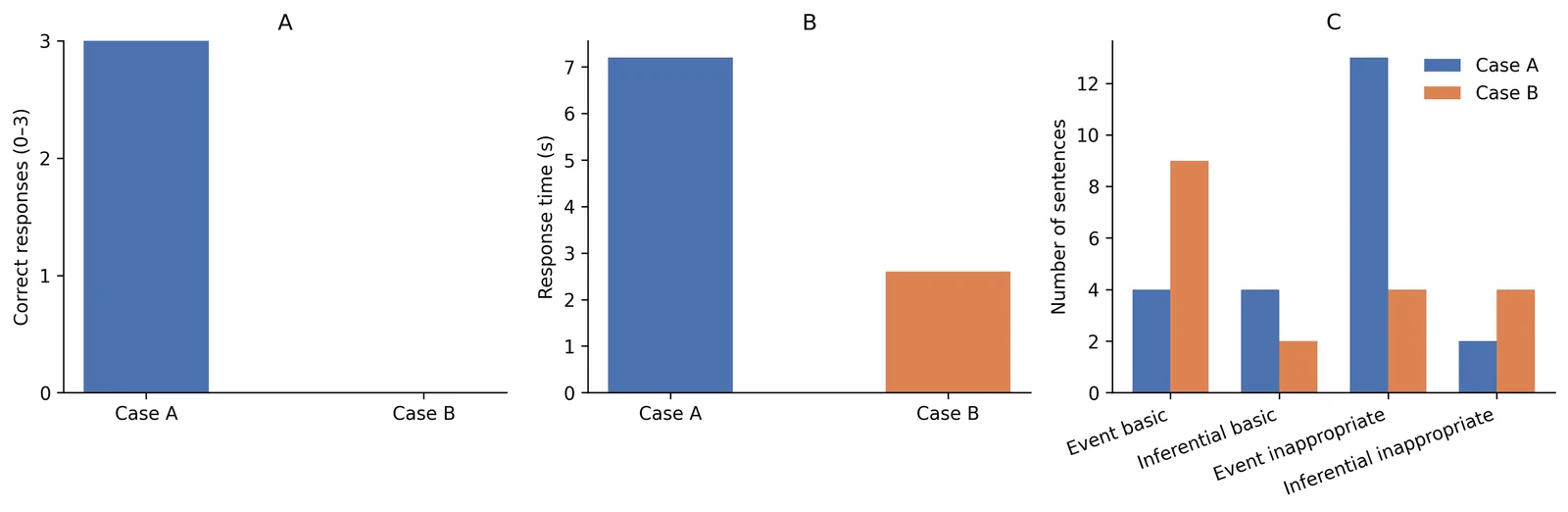
Discourse characteristics and theory of mind performance in the two cases (A) Accuracy on the theory of mind task, (B) response time, and (C) distribution of basic and inappropriate sentences in event-related and inferential discourse

Discourse performance was evaluated using a four-frame cartoon description task. All utterances were transcribed verbatim and classified as event-related or inferential statements. Each statement was further categorized as a basic sentence or an inappropriate sentence based on discourse appropriateness and coherence [15]. ToM was assessed using the Strange Stories test developed by Happé [6], which evaluates the ability to infer characters’ intentions and beliefs beyond literal meanings. On the ToM task, Case A demonstrated intact performance, whereas Case B showed marked impairment. Response time on the ToM task was shorter in Case B than in Case A. Analysis of discourse structure revealed qualitative differences between the two cases. Case A produced a greater number of inappropriate sentences in event-related statements but demonstrated a relatively high number of appropriate inferential statements. In contrast, Case B showed relatively preserved accuracy in event-related statements but a higher proportion of inappropriate inferential statements. These results indicate a dissociation between event representation and inferential discourse processing across the two cases (Figure 2).

## Discussion

The present case series highlights qualitative heterogeneity in discourse impairment following RHD. Although both patients had right hemisphere lesions, the nature of their discourse difficulties differed substantially, suggesting that discourse impairment after RHD does not arise from a single, uniform mechanism [16,17]. In Case A, discourse impairment was characterized primarily by errors in event-related statements, whereas inferential discourse was relatively preserved. This profile suggests difficulty in organizing or selecting salient event information rather than a fundamental impairment in mental state reasoning. Previous studies have indicated that anterior regions of the right hemisphere contribute to discourse organization, executive control, and the global structuring of narratives. Disruption of these processes may result in fragmented or inappropriate event descriptions while sparing inferential understanding [16,18]. In contrast, Case B demonstrated marked impairments in inferential discourse accompanied by deficits on the ToM task. Inferential processing is essential for understanding implied meaning, speaker intentions, and causal relationships within discourse. Previous studies have shown that right hemisphere damage may be associated with impairments in inferential discourse processing and mental-state attribution [11,16,19]. The co-occurrence of inferential discourse errors and ToM deficits in Case B may therefore reflect overlapping higher-order inferential and social-cognitive processes. Because lesion extent and network involvement were not systematically quantified in the present case series, we do not infer a specific neuroanatomical substrate for these deficits. Importantly, both cases shared right hemisphere involvement but differed in lesion characteristics and qualitative discourse profiles. This dissociation supports the view that discourse impairment after RHD reflects the involvement of multiple interacting neural systems. Previous studies have likewise demonstrated heterogeneous communication profiles after RHD and impairments across multiple components and levels of discourse processing [14,16,17]. The present cases extend these findings by providing clinically grounded examples illustrating how different components of discourse, event representation versus inferential reasoning, may be differentially affected. From a clinical perspective, these findings underscore the importance of qualitative discourse analysis rather than reliance on global severity measures alone. Although both patients would be classified as having discourse impairment, the nature of their difficulties differed substantially, with distinct implications for assessment and intervention. In particular, inferential discourse deficits associated with ToM impairment, as observed in Case B, may require targeted evaluation of social-cognitive functions, whereas event-related discourse difficulties, as seen in Case A, may benefit from interventions focusing on narrative organization and executive support. Several limitations should be acknowledged. This case series includes only two patients, and the findings cannot be generalized without caution. In addition, lesion extent and network involvement were not systematically quantified. Nevertheless, detailed qualitative comparison provides valuable insights that complement group-based studies and highlights mechanisms that may be overlooked by quantitative measures alone. Future research should examine larger cohorts of patients with RHD using combined qualitative discourse analysis, standardized social cognition measures, and lesion-symptom mapping. Such approaches may further clarify how distinct right hemispheric networks contribute to different components of discourse and inform more individualized clinical assessment and rehabilitation strategies.

## Conclusions

This case series demonstrates that discourse impairment following RHD may present with qualitatively distinct profiles. In particular, inferential discourse deficits may be associated with ToM impairment in some patients but not in others. These findings highlight the clinical value of qualitative discourse analysis for individualized assessment.
