# Correction: Relationship Between Qualitative Differences in Discourse Impairment and Theory of Mind in Patients With Right Hemisphere Damage: A Case Series 

**DOI:** 10.7759/cureus.c477

**Published:** 2026-09-04

**Authors:** Kengo Hoyano, Yasutaka Kobayashi

**Affiliations:** 1 Graduate School of Health Sciences, Fukui Health Science University, Fukui, JPN

This article has been corrected following a reader inquiry and subsequent editorial review. Several references that did not support the statements citing them, or that could not be verified, have been replaced or removed, and the reference list has been revised and renumbered. Two sentences in the Discussion have been revised accordingly. The authors have additionally disclosed the use of OpenAI's ChatGPT for literature-search assistance and English-language editing, which was not stated in the original submission; an AI-use disclosure has been added to the article. The Abstract, case data, results, and Conclusions are unaffected. The authors apologize for the errors.

